# Nurses' perceptions of their supportive role for cancer patients: A qualitative study

**DOI:** 10.1002/nop2.1112

**Published:** 2021-11-03

**Authors:** Mostafa Bafandeh Zendeh, Masumeh Hemmati Maslakpak, Madineh Jasemi

**Affiliations:** ^1^ Department of Critical Care Nursing Students' Research Committee School of Nursing and Midwifery Urmia University of Medical Sciences Urmia Iran; ^2^ Faculty of Nursing and Midwifery Urmia University of Medical Sciences Urmia Iran

**Keywords:** cancer patient, content analysis, nurses, nurses' perceptions, patient advocacy

## Abstract

**Aim:**

Supporting cancer patients is one of the integral, ethical and professional components and concepts of nursing care. Given the prominence of nurses' understanding of their supportive role in providing quality and humane nursing care for cancer patients, it is crucial for them to acquire sufficient knowledge to achieve a positive attitude towards patient support.

**Design:**

Qualitative conventional content analysis approach was used.

**Methods:**

This study was conducted with a qualitative approach and conventional content analysis in 2020. Participants consisted of 18 nurses of different oncology wards of teaching hospitals in Northwestern Iran recruited using the purposive sampling method. Data were collected through semi‐structured interviews and analysed simultaneously with data collection (22 july ‐ 20 june 2020).

**Findings:**

The analysis of interviews showed that the main theme of “a canopy as a supportive role for cancer patients” was formed. In this theme, related subcategories included patient's psychological support (compassionate care with emotional support, having an intimate/friendly relationship with the patient and communicative behaviour facing patient needs), patient training (need‐based training, having good theoretical knowledge about the patient and having a role model for playing a supportive role for the patient) and supporting the patient with clinical self‐efficacy (being responsible with clinical competency, understanding the patient's behaviour and attempt to provide extra‐duty care).

## 1 INTRODUCTION

Cancer is a debilitating and threatening disease and one of the global health problems with increasing prevalence (Zamanzadeh et al., 2013). A cancer diagnosis is an extremely unpleasant and unbelievable experience for individuals, and they often face a type of inner turmoil, identity conflict, isolation, anxiety, and depression, have difficulty in communicating with others (Monterosso et al., 2019), and need psychological and social‐emotional support to increase self‐confidence and a sense of peace (Nielsen et al., 2017). Support is one of the effective sources to control cancer and resulting complications (Haisfield‐Wolfe et al., 2012). Studies have shown that lack of support has negative consequences on patients (Black, 2011; Vitale et al., 2019).

Supporting the patient is an inherent sense of professional nursing. This ethical principle is necessary for the nurse–patient relationship and reflects respect for patients as human beings and their rights (Jafarian Amiri et al., 2020). Therefore, supporting patients as an essential part of the nursing profession is defined as encouraging and protecting patients' health and interests, providing them with information, assisting clients in making decisions, patient independence and empowerment, supporting the client from unnecessary concerns, respecting patients' values and beliefs along with training and communicating with the patient (Nsiah et al., 2019). Supporting basic issues is the foundation of nursing and exclusively reflects the nature of nursing care (Vaartio‐Rajalin& Leino‐Kilpi, 2011). Furthermore, support as the “philosophical basis and purpose of nursing” has an effective role in the patient's self‐control in determining their destiny (Scott, 2017).

Psychological support by establishing appropriate therapeutic communication between nurses and cancer patients in addition to making decisions and involving patients in the care process and also preventing the side effects of cancer treatments during the illness, especially in hospitalized patients, is necessary (Franklin et al., 2019), and it exchanges the useful information and changes negative emotions and moods to positive and hopeful emotions (Hack et al., 2012). Therefore, as one of the first health care providers who work closely with patients, nurses must learn how to use this supportive‐communicative mechanism for these patients (Kiani et al., 2016). According to the articles, patient vulnerability requires more ethical principles in nursing care (Nsiah et al., 2019).

According to the importance of supporting cancer patients, the role of nurses in this field is of importance, and one of the factors in this area is nurses' perception of how to play a supportive role (Nsiah et al., 2019). It seems that what oncology nurses know or understand about supporting patients in clinical practice in Iran is far from its true meaning. On the other hand, the evidence shows that the type of nurses' perception of being supportive determines how they should defend their patients (Abdolrahimi et al., 2017). Therefore, it is essential to carefully examine how nurses perceive and describe their supportiveness in clinical settings, have a deep understanding of the phenomenon of supporting cancer patients and identify interactions in this case. These interactions are beneficial for expanding communication methods and improving care quality. A qualitative approach can display the extensive dimensions of this phenomenon to researchers and help them gain a better understanding of the phenomenon. This approach is based on participants' actual experiences and achieves more realistic results(Sherko et al., 2013). In recent decades, in different countries, especially in Iran, limited qualitative studies have been conducted to institutionalize the concept of support (Jafarian Amiri et al., 2020; Motamed‐Jahromi et al., 2017). However, these studies have not addressed the role of communication in interactive, subjective and dynamic components of supporting cancer patients; therefore, this study aims to examine the nurse's perception of playing a supportive role for cancer patients.

## 2 METHOD

### 2.1 Aim and design

In this study, a qualitative method with a content analysis approach was used. The above method is based on inductive reasoning and has three acceptable or conventional, directed and summative approaches (Wildemuth, 2016). In this study, the conventional approach was used to describe the nurses' experiences of supporting cancer patients. This method interprets the textual content of the data and describes and organizes the phenomenon by a systematic coding process and identifying themes or patterns (Armat et al., 2018).

This study was conducted in the main oncology centres of northwest Iran. The selected hospitals for this study are the main treatment and referral centres for cancer patients in the northwest of Iran. Since oncology centres in Iran and, consequently, in northwestern Iran are centralized in the capital of provinces, cancer patients are referred to a subspecialty hospital in these cities. The capital of provinces are the best centres with medical facilities for oncology patients; therefore, the researcher has chosen these two centres in the northwest. Besides, considering that the researcher's study place is in West Azerbaijan province with Urmia as its capital city, he has performed sampling for the data richness. In order to perform theoretical sampling, some participants were selected from the neighbouring province, East Azerbaijan, with Tabriz as its capital, and interviewed.

Support is the social interaction process that first arises by establishing empathetic communication and building a safety net for the nurse‐patient system. The aim of this study was to demonstrate nurses' professional power by acquiring this capability, which is a feature that helps them therapeutically communicate with cancer patients. Nurses perform it by taking a supportive role for the patient.

### 2.2 Participants

Qualified participants were identified through face‐to‐face conversations with nurses and head nurses in teaching hospitals affiliated to Urmia and Tabriz University of Medical Sciences in West and East Azerbaijan provinces.

This study was conducted with 18 participants, including seven men and ten women with a mean age of 35 years and an average work experience of 12 ± 23 years, whose characteristics are listed in Table 1. At first, experienced nurses having a supportive role were selected. In the purposive sampling stage, participants were selected based on their perspectives towards the phenomenon. Afterward, according to the interview results and in order to confirm the findings, the theoretical sampling method was used. The sampling process was continued until data saturation was reached. When the data were repetitive, and no new codes were identified, sampling was stopped. An attempt was made to diversify and enrich the sampling process and to have a comprehensive view of the phenomenon. Participants were selected from diverse gender, levels of education and cultural backgrounds.

**TABLE 1:** Demographic characteristics of the participants

No.	Education	Work experience	Position	City
1	Bachelor's	22	Clinical nurse	Urmia
2	Master's	18	Faculty member	Urmia
3	Bachelor's	15	Clinical nurse	Urmia
4	Bachelor's	25	Clinical nurse	Urmia
5	Bachelor's	3	Clinical nurse	Tabriz
6	Bachelor's	6	Clinical nurse	Tabriz
7	Bachelor's	12	Clinical nurse	Urmia
8	Bachelor's	21	Clinical nurse	Tabriz
9	Bachelor's	9	Clinical nurse	Tabriz
10	Bachelor's	13	Clinical nurse	Tabriz
11	Bachelor's	6	Clinical nurse	Urmia
12	Master's	21	Clinical nurse	Urmia
13	Bachelor's	8	Clinical nurse	Urmia
14	Bachelor's	10	Clinical nurse	Urmia
15	Master's	13	Clinical nurse	Tabriz
16	PhD	6	Faculty member	Urmia
17	Bachelor's	4	Clinical nurse	Tabriz
18	Bachelor's	8	Clinical nurse	Tabriz

### 2.3 Data collection

Data were collected through semi‐structured and face‐to‐face interviews over six months from July to February 2020. Interviews were conducted at the time and place determined by the participants (mainly in the head nurse's room or the hospital conference room during break hours with coordination) after informed consent was obtained from them. Each interview lasted 30–90 min. Due to participants' fatigue, the time was arranged, and the interview was postponed to another time if necessary.

Eighteen interviews were conducted and analysed by a research team (MJ and MB) qualified to conduct qualitative interviews. In four cases, subsequent interviews were conducted in order to get further information.

Participants were asked about their experiences of supporting patients. According to the abstract nature of the subject, the researchers represented more objective questions. In the beginning, participants were asked general questions to reduce stress and increase their confidence. Then, the main semi‐structured questions were asked, such as “What supportive experiences do you have, and what are the effective factors of supporting patients?” If it's possible, give examples of your actual experiences in this field. How was your feeling in these situations? “What does it mean to support patients?” With participants' permission, the interviews were audio‐recorded and transcribed verbatim for accurate implementation. Subsequently, based on participants' responses, the interviews became more structured, focusing on supporting patients, such as “How do you support cancer patients?” The researchers continued the interviews until the data saturation was achieved; in other words, no new idea, concept or category emerged from the final interviews (three consecutive interviews).

In order to have better communication with the study environment and participants and analyse the actual data, researchers used field notes. In this study, field notes also provided the opportunity to confirm participant's psychological and emotional reactions immediately after the interview. For example, attending the oncology ward of a hospital in Urmia and observing how nurses communicate with cancer patients lead to a note that focuses on the supportive role in clinical care, reflecting the consequences of this type of communication and confirming communicational behaviour between nurses and patients.

### 2.4 Data analysis

Data collection and analysis were performed simultaneously. Data were analysed using Grundheim and Lundman's (2004) technique. In this method, a complete interview is considered a unit of analysis, including notes that must be analysed and coded (Graneheim & Lundman, 2004). We used the following steps to generate codes through direct and inductive data evaluation: The researchers listened to the interviews several times and transcribed the recorded interviews word by word using MAXQDA software Ver 10. Paragraphs, sentences and words were considered meaning units. A meaning unit is a set of words and sentences related to each other in terms of content and categorized based on their context and content. The texts were reviewed several times to highlight words containing key concepts or meaning units and to extract the initial codes. The codes were checked several times in a continuous process, from code extraction to tagging. Similar codes were merged, categorized and tagged, and subcategories were identified. The extracted subcategories were eventually compared and (if possible) merged to form the main categories. Eventually, three subcategories were formed, leading to the emergence of the main theme.

### 2.5 Trustworthiness

Lincoln and Guba (1986) criteria were used to confirm the accuracy and stability of research data. The credibility of the data was confirmed by using member‐checking and prolonged engagement techniques. In order to verify the findings by the participants and ensure the accurate and true reflection of their experiences, the content of the interview and the resulting codes were reviewed. The data were also assessed by an external researcher (peer‐checking). To ensure reliability and dependability, the methods of data collection, interviewing, taking notes, coding and analysing the data were thoroughly reviewed and judged by an external auditor (external audit). The audit method was used to achieve conformability so that all stages of the research, especially the stages of data analysis and results, were presented, and two colleagues reviewed them in the field of qualitative research. The transferability of the findings was also demonstrated by providing a detailed description of the research report and the content of the interviews with selected quotes from the participants. Using field notes during the interview also enabled the researcher to check the accuracy of the content while analysing the data (Lincoln & Guba, 1986).

## 3 RESULTS

According to the results, the categories of “providing psychological support,” “patient training (supporting by information)” and “patient support with clinical self‐efficacy” led to the emergence of the main theme called “Being a Canopy for cancer patients” (Table 2).

**TABLE 2:** Categories, subcategories and codes extracted from the interview analysis

Core category	Subcategories (1)	Primary concepts	Open codes
Being a Canopy for cancer patients	Providing psychological support	Caring compassionately with emotional support	Giving spiritual‐emotional support along with a sense of hope to the patient Giving an optimistic view of the future by reassuring explanation to the patient‐
		Having an intimate/friendly relationship with the patient	Having warm and kind behaviour by nurses‐ Being friendly with patients
		Having communicative behaviour facing patient needs	Allowing the patient to express emotions ‐ dealing appropriately with the patient's condition
	Information advocacy and patient training	Need‐based training	Increasing the patient's information about his/her illness Teaching self‐care methods at home
		Having theoretical knowledge about the patient	Guiding cancer patients‐ Being an information reference
		Having a role model for playing a supportive role for the patient	Being a role model for colleagues through the communicative behaviour with the patient ‐ Inviting beginners to be patient in stressful cases
	Supporting the patient with clinical self‐efficacy	Being responsible with clinical competency	Having the ability and competence to care for the patient ‐ establishing a relationship between the nurse and the patient while performing clinical care
		Understanding patient's behaviour	Predicting the patient's condition with the help of nurses' work experience ‐ considering the consequences in dealing with the patient
		Attempt to deliver care beyond the routines	Making attempts beyond routine for the patient benevolently ‐ Sharing the patient's personal problems with the nurse

### 3.1 Being a Canopy for patients suffering from cancer

Oncology nurses have various simultaneous roles, especially supportive roles. The data indicated that nurses with clinical self‐efficacy advocated their patients by providing and increasing information about patient's needs and preferences about the health care system, and finally, they supported patients psychologically and gave them a great canopy. These items were considered as subsets of this topic.

#### 3.1.1 Having psychological support

Oncology nurses improve their patients' interests by analysing psychological and physical distresses and performing psychological care programs for patients, especially at the beginning of the disease. In this study, participants identified some of the characteristics of psychological support: caring compassionately with emotional support, having an intimate/friendly relationship with the patient and having communicative behaviour facing patient needs. These cases highlight communicative behaviour between the nurse and patient.

##### Caring compassionately with emotional support

Giving encouraging and reassuring explanations and listening to the patients along with positive feedbacks are the main factors of the compassionate‐emotional caring process. Participants strongly emphasized that “All patients usually need to talk, and you should have enough patience to hear and give this positive message and a green light to the patients, and consider that if you answer her/his questions with patience, the patient will feel better” (M‐7).

The participants' statements indicated that they described the emotional support of the anxious patient with warm behaviour and jokes, which creates “a sense of hope.” In this regard, one of the participants stated:

The first time that the patient comes to the ward, he/she is in a state of shock and denial due to his/her illness. We try to treat and behave him/her more kindly and emotionally than the others. When patients see that we joke and laugh with them, they forget those feelings and feel better. (M‐1)

##### Having an intimate /friendly relationship with the patient

One of the main features of psychological support is the sense of empathy with friendship and intimacy that nurses have toward the patients. Participants mentioned issues such as warm and intimate behaviour, concurrence (physical and emotional presence) and comforting the patient by being close to them. They also argued that these cases were comforting the patients. One of the participants stated:

I try to be close to the patient and behave like a friend, so they feel comfortable with me. (M‐9)

Another participant stated: “while working, I try to put myself in the patient's shoes; I try to understand his/her expectations. This understanding is enough for the patient, and he/she feels calm. I try to behave in a way that is pleasant for the patient.” (M‐4).

##### Having communicative behaviour facing patient needs

Building a therapeutic relationship in a supportive‐social environment needs to fulfill patients' expectations by respecting them and strengthen their self‐confidence to reduce their stress. The results showed that allowing the patient to express his/her feelings and nurses' suitable encounter creates a good feeling in the patient. A participant stated:

Because of the chronic nature of their disease, these conditions occur, and they suffer the pain caused by the disease and treatment measures. It causes emotional problems. They have higher expectations and needs than other patients. They expect that nurses pay more attention to them and support cancer patients more than other patients. (M‐11)

A participant stated:

My initial behaviour with cancer patients as a nurse has a great impact on their ability to express themselves and lead to self‐control. (M‐8)

#### 3.1.2 Informational advocacy with patient training

Oncology nurses should educate patients about cancer during the disease period before they start their treatment. In this regard, the results showed that features such as need‐based education for patient self‐care, nursing resourcefulness with good theoretical knowledge in responding to the patient, and having a role model for playing a supportive role for the patient are the strategies that Iranian oncology nurses apply in order to educate and increase patients' awareness.

##### Need‐based education for patient self‐care

Based on the participants' experiences in this study, one of the most significant themes that refer to the patient's concerns is the lack of knowledge about the disease and the process of treatment and care. Therefore, they make an effort to provide the necessary training and explanations to increase patients' information and respond to their needs, based on their needs and questions. A participant said:

I try to relieve the patient's concerns by increasing his or her knowledge about that disease. (M‐6)

Another participant stated in this regard:

Being a nurse isn't just giving medicine. I try to educate patients to learn the care process because they need to take care of themselves at home, and this training will reduce their problems after discharge. (M‐10)

##### Nursing resourcefulness with good theoretical knowledge in responding to the patient

Since nursing is a practical system based on professional knowledge, it is necessary to use it as a reference to better respond to patients' treatment questions. Most of the participants believe that the role of the nurse's rich knowledge in guiding/supporting patients is evident. In this regard, participant No. 5 stated:

Working in the oncology ward has a great duty and responsibility for the nurse to constantly study and increase his/her information and to understand his/her duty as a nurse, which is important and increases the nurses' duty in updating and increasing their knowledge. (M11)

Another participant mentioned:

When a patient asks a question, and I answer openly, it creates a sense of trust in her/him, so he/she comes to me whenever they need information about the disease and treatment. (M‐15)

##### Having a role model for playing a supportive role for the patient

Some participants mentioned this case as a role model for beginners (since in the first days of working with the patient, they had problems and tensions) in teaching and advising the patient. One participant argued that by comforting beginners, they could believe in their abilities and use this method as a model for reducing stress and gaining their caring ability.

Certainly, there were initial stresses about communicating with these patients. Will it be easy to communicate with them? What problems, needs, and concerns do these patients have? Will we be able to meet their needs? “As an experienced nurse, I try to be a good role model for giving information to these colleagues who gradually learn and become aware of their duty.” (M‐14)

#### 3.1.3 Supporting the patient with clinical self‐efficacy

Codes extracted from these interviews led to the emergence of three characteristics: competency for caregiving and responsibility, responsiveness along with increasing clinical experience, and the ability to recognize the patient's behaviour by empirical background and performing extra‐duty tasks. The relationship between these three characteristics led to the emergence of the category “patient support with clinical self‐efficacy in nurses”, revealing the fact that this helps nurses observe the patient's rights, provide quality care and promote the interests of their patients. Self‐efficacy to perform a clinical role is a psychosocial strategy for supportive care.

##### Caring competency and being responsible: responsiveness is gained through increasing clinical experience

“The commitment to provide the best care to cancer patients leads to the prioritization of patient care, which is achieved by communicating with the patient along with performing clinical care.” Defining responsiveness to the patient is possible through a sense of responsibility. This sense is evident with features such as increasing work experience, professional competence and accuracy in performing procedures by developing knowledge.

The sense of responsibility in caring for these patients is very important. I myself try to be one of those people who do the patient's work correctly; for example, I try to be available all the time. I feel that now I'm paying more attention to this issue than a few years ago. (M‐12)

Of course, the oncology nurse must be scientifically and educationally qualified to provide service. Recently, a certificate of clinical and professional competence has been issued. So the person who receives this certificate is specialized in this work. Nursing, treatment, knowledge, and practical skills are important, which can be increased by experience. (M‐8)

##### Ability to recognize the patient's behaviour with empirical background

Nurses stated that they could predict the patient's behavioural status and needs over time by working in the ward, dealing with oncology patients and gaining work experience.

I know that as soon as these patients enter the ward, they're kind of confused because of the disease and its consequences and show aggressive behaviours. I try to understand their condition and treat them appropriately. (p7)

Other participants found backgrounds as a factor in communicating appropriately with these patients.

The bad events of the past have been an eye‐opening experience for me and have given me the necessary awareness, especially about end‐of‐life care, that I mustn't be indifferent to the needs of this situation and try at least to reduce patients' pain. (p17)

##### Attempt to deliver care beyond the routines

Nurses who participated in the interview stated that they sometimes performed tasks for the patient or their family empathically that were not within their scope of responsibility; however, a sense of humanity encouraged them to take such actions, called “extra‐duty” actions. These nurses believed that no one could interrogate them for not performing these extra tasks; however, their intrinsic motivations led them to perform these actions.

Supportive care is done along with the support process. In addition to meeting the patient's needs, which is part of my legal duties, I can do things beyond that for the patient that are not part of my job. (M‐13)

## 4 DISCUSSION

Based on the findings, psychological support, informational advocacy with patient training and the patient's self‐efficacy were identified and introduced as nurses' efforts to support cancer patients. These factors were introduced as the main categories and subcategories of research in this study.

One of the most important aspects reported in this study is the psychological support of cancer patients, by which the nurse helps the patient solve problems and meet his/her needs. These cases are mentioned in other studies (Nielsen et al., 2017; Vaartio‐Rajalin & Leino‐Kilp, 2011). Based on the participant's experience, one of the examples of psychological support is emotional support with compassionate care. According to Hofferman and Lane (2017), in order to support patients and their families, the nurse should help him/her get along with conditions and respect the patient's beliefs, which are included as compassionate care. It is evident that the treatment process is not sufficient, and the medical staff need to provide emotional support, empathy and compassion so that patients can adapt to their treatments, anxieties and fears. Therefore, the findings of the study show that in order to have a supportive role, the nurse should behave sympathetically. In this regard, Post et al. (2014) found that sincere presence at the patient's bedside is more effective than most medications, reduces anxiety and facilitates their recovery process. According to participants, this issue affects the nurses' communicative behaviour facing patients' needs. Ghafourifard et al. (2017) argued that a good and more effective relationship must be established between the patient and the nurse; consequently, nurses will be able to understand patients' sufferings. In this regard, Power (2016) likewise stated that communicating with the patient, receiving feedback and sympathetic relationships are essential indicators of care quality.

By identifying the patient's needs, sharing and arranging the information, nurses can guide the patient and help him/her control the disease; therefore, the uncertainty level will decrease. This kind of support begins at the onset of the disease, and patients are guided through questions in the form of information‐seeking behaviours by professional sources, especially nurses. It is similarly mentioned in other studies (Abbasinia et al., 2020; Hack at al., 2012), and findings showed that nurses highlighted this type of experience. In this regard, need‐based training is considered a facilitator of the patient's self‐care, and it is believed that most patients are concerned about their lack of knowledge about the disease and the process of treatment and care. Therefore, nurses attempt to train patients according to their questions and conditions and increase their self‐care knowledge. Arman and Hogg (2016) found that individuals, such as cancer patients, who suffer a lot, desire to care for themselves. To do this, they need the help of medical staff to teach them how to take care of themselves so that they can have a meaningful and good life. Participants reported that responding to the needs of these patients and being a professional reference with good theoretical knowledge are the duties of a clinical trainee of oncology wards. Goetz et al. (2010) argued that the initial treatment counseling given to oncology patients was an important factor of the patient's satisfaction and trust, and this type of supportive counseling, while reducing patients' anxiety, provided confidence to the medical staff. Participants stated that colleagues and other nurses act as role models to have an educational, supportive role, and they try to emulate the behaviour of supportive nurses. In her study, Pucino (2014) highlighted the importance of role models in the development of supportive behaviours influenced by compassionate clinical. She believed that young nurses consider experienced staff as their role models.

Nurses' self‐efficacy in performing their clinical role is the main psychological strategy for providing supportive care to cancer patients. This helps nurses to observe patients' rights and provide quality and competent care. Jasemi et al. (2019) argued that empowering oneself for a clinical role is a psychosocial‐social strategy for conscience‐based care. In his study, Akpotor and Johnson (2018) argued that the self‐efficacy of oncology nurses in a social‐supportive environment is the best way to establish a nurse–patient therapeutic relationship that improves confidence and satisfaction with the care services, which are shown in the findings of interviews. This self‐efficacy is effective in nursing activities, leading to an increase in caring competence, responsibility and level of accountability (Vaartio‐Rajalin & Leino‐Kilpi, 2011). This promotion helps nurses extend their recognition power and establish a good therapeutic relationship with the patient (Abbasinia et al., 2020). Participants' experiences have ensured that. This interaction motivates the nurse to make an attempt beyond the routines, evaluate the patient and recognize his/her needs (Jasemi et al., 2019; Rahmani et al., 2017).

## 5 CONCLUSION

Developing a strategy is useful to increase the support for cancer patients and improve safety and the quality of nursing care in the health care system. Counseling and responsiveness of nurses as advocates of cancer patients should be further investigated and promoted in oncology nursing care. Therefore, it is necessary to make coordinated efforts in the education, research and nursing management fields to professionalize this role and help nurses perform their critical role as patient supporters. Empowering oncology nurses is the best way to establish a therapeutic relationship between nurse and patient.

### 5.1 Limitations

The findings of this study are limited to the perception of nurses' supportive role in oncology wards in Iranian culture. Supporting cancer patients requires further studies with quantitative and qualitative approaches in different cultures. Including only two teaching hospitals in Iran was another limitation of this study. Likewise, it may not represent the experiences of all members of the oncology nursing profession in Iran. Limitations of our study indicate the need for further studies with larger and hybrid groups in different cultures.

## 6 IMPLICATION FOR NURSING

Clinical empathy as an effective strategy can help support these patients. It can be an opportunity for counseling activities and nurses' responsiveness as cancer patients' supporters in oncology nursing care.

## CONFLICT OF INTEREST

The authors declare that they have no competing interests.

## AUTHOR CONTRIBUTIONS

MJ: design the study, interpretation of data, and writing the paper; MHM: performing the focus group and writing the paper; MB: collection, analyse and interpretation of data and writing the paper.

## ETHICAL APPROVAL

This study was approved by the ethics committee of Urmia University of Medical Sciences (Code: IR.UMSU.REC.1398,288). The letter of introduction was presented to the hospitals upon attendance. In the field, the purpose of the study, the method of interview and the rights of the participants were explained to all nurses, and their informed written consent, which allows the researchers to use the data for research, was acquired.

## Data Availability

Researchers allow this article to be used by anyone interested. All data generated during this study are included in this published article.
